# Δ-6 Desaturase Substrate Competition: Dietary Linoleic Acid (18∶2n-6) Has Only Trivial Effects on α-Linolenic Acid (18∶3n-3) Bioconversion in the Teleost Rainbow Trout

**DOI:** 10.1371/journal.pone.0057463

**Published:** 2013-02-27

**Authors:** James A. Emery, Karen Hermon, Noor K. A. Hamid, John A. Donald, Giovanni M. Turchini

**Affiliations:** 1 School of Life and Environmental Sciences, Deakin University, Warrnambool, Victoria, Australia; 2 School of Life and Environmental Sciences, Deakin University, Waurn Ponds, Geelong, Victoria, Australia; Rikagaku Kenkyūsho Brain Science Institute, Japan

## Abstract

It is generally accepted that, in vertebrates, omega-3 (n-3) and omega-6 (n-6) poly-unsaturated fatty acids (PUFA) compete for Δ-6 desaturase enzyme in order to be bioconverted into long-chain PUFA (LC-PUFA). However, recent studies into teleost fatty acid metabolism suggest that these metabolic processes may not conform entirely to what has been previously observed in mammals and other animal models. Recent work on rainbow trout has led us to question specifically if linoleic acid (LA, 18∶2n-6) and α-linolenic acid (ALA, 18∶3n-3) (Δ-6 desaturase substrates) are in direct competition for access to Δ-6 desaturase. Two experimental diets were formulated with fixed levels of ALA, while LA levels were varied (high and low) to examine if increased availability of LA would result in decreased bioconversion of ALA to its LC-PUFA products through substrate competition. No significant difference in ALA metabolism towards n-3 LC-PUFA was exhibited between diets while significant differences were observed in LA metabolism towards n-6 LC-PUFA. These results are evidence for minor if any competition between substrates for Δ-6 desaturase, suggesting that, paradoxically, the activity of Δ-6 desaturase on n-3 and n-6 substrates is independent. These results call for a paradigm shift in the way we approach teleost fatty acid metabolism. The findings are also important with regard to diet formulation in the aquaculture industry as they indicate that there should be no concern for possible substrate competition between 18∶3n-3 and 18∶2n-6, when aiming at increased n-3 LC-PUFA bioconversion *in vivo*.

## Introduction

Due to economic and environmental concerns shaping the rapidly expanding aquaculture sector, marine fish oil in fish feed (aquafeed) formulations is being increasingly reduced and substituted with alternative oils of terrestrial origin, such as vegetable oils and animal fats [1]–[3]. The direct result of this shift in feed formulation is that present aquafeeds contain progressively lesser long chain polyunsaturated fatty acids of the omega-3 series (n-3 LC-PUFA), which are known to deliver health benefits to humans consuming seafood [4]–[6]. Ultimately, this results in a detrimental modification of the final content of these health-promoting fatty acids in the flesh of cultured fish [7], [8]. In an attempt to overcome this problem, the understanding of the *in vivo* n-3 LC-PUFA biosynthetic capability of fish is attracting significant research effort, and it is envisaged that increased knowledge in this area will contribute towards the long term sustainability of the entire fisheries and aquaculture sector [9].

In all vertebrates, the *in vivo* n-3 LC-PUFA biosynthetic pathway is conceptually identical [10], [11], and involves a series of enzymatic steps catalysed by two fatty acid desaturase enzymes (Δ-6 desaturase and Δ-5 desaturase), two fatty acid elongase enzymes (Elovl-5 and Elovl-2) and a peroxisomal β-oxidation for fatty acid chain shortening [12], [13]. This pathway, commonly referred to as the “Sprecher pathway”, can convert the two essential polyunsaturated fatty acids (PUFA) linoleic acid (LA, 18∶2n-6) and α-linolenic acid (ALA, 18∶3n-3) into n-6 LC-PUFA (such as arachidonic acid, ARA 20∶4n-6), and n-3 LC-PUFA (such as eicosapentaenoic acid, EPA 20∶5n-3, and docosahexaenoic acid, DHA 22∶6n-3), respectively. Because of evolution and adaptation to the environment, each species has a different capacity for bioconversion of PUFA into LC-PUFA, depending on the presence, abundance and activity of the specific enzymes in the metabolic pathway [11], [14]–[16]. For example, it can be argued that the ability of biosynthesising n-3 LC-PUFA is greater in fish compared to birds, and greater in birds compared to humans, further emphasizing the importance of aquaculture as a provider of nutritional n-3 LC-PUFA [4], [15], [17], [18]. Recently, variants in the fatty acid desaturase gene cluster that result in different enzyme efficiencies and adaptive evolution have been documented in humans [19]. Similarly, differences in the efficiency of this metabolic activity amongst closely related species have been reported. For example, within teleosts there are species such as salmonids that are capable of bioconverting n-3 LC-PUFA, and other species such as several top-order marine predators that seem to have lost this capability [11].

Much of our knowledge of *in vivo* fatty acid metabolism originates from studies on mammals (primarily rodents), but in recent years many advances have been made in understanding fatty acid metabolism in fish, and some peculiarities have been reported [20]–[23]. However, some of the paradigms proposed for mammals are also valid for fish. Of high relevance are the early pioneering studies on LA and ALA bioconversion in rats by Holman’s group [24], [25], which led to the acceptance that the same enzymes are involved in the biosynthesis of n-3 and n-6 PUFA, respectively. This was confirmed by a series of later studies [26], [27] and led to the well-accepted paradigm that the two substrates (LA and ALA) are in direct competition for accessing Δ-6 desaturase (the first of the bioconverting enzymes). However, in a recent review of the fatty acid Δ-6 desaturase in teleosts [28], it was reported that the data on Δ-6 desaturase substrate competition in appear to be somewhat contrasting, and the authors urged further investigation to better identify these mechanisms.

Rainbow trout (*Oncorhynchus mykiss*) are one of the most commonly cultured salmonids, and their farming is responsible for a large use of fish oil [29]. Thus, finding possible remedial strategies towards minimising the use of this commodity, while at the same time maintaining optimal nutritional quality of the final product, is a highly relevant and timely objective [9]. We have recently shown the following in trout: i) they are capable of efficiently bioconverting ALA up to EPA and DHA [18]; ii) the elimination of dietary n-3 LC-PUFA (enzyme products) up-regulates the transcription rate of Δ-6 desaturase mRNA; however, the total apparent *in vivo* enzyme activity does not correlate with Δ-6 desaturase mRNA expression [30]; iii) that the LC-PUFA biosynthetic pathway is substrate limited [31]; and iv) that the provision of increased dietary stearidonic acid (18∶4n-3), over ALA, has only minimal benefit in terms of total n-3 LC-PUFA biosynthesis, suggesting that Δ-6 desaturase cannot be considered as the rate-limiting step in this pathway [32].

Thus, in light of the differences reported in the fatty acid bioconversion metabolism of teleosts compared to that of mammals, the aim of this study was to directly assess if the commonly accepted paradigm that the two substrates (LA and ALA) are in direct competition for accessing Δ-6 desaturase was valid for fish as it is in mammals. To achieve this, two triplicate groups of rainbow trout were fed two experimental diets characterised by identical dietary ALA but different LA content. The *in vivo* fatty acid metabolism, and in particular, the bioconversion of ALA towards n-3 LC-PUFA was then assessed. The results from this study are envisaged to be also directly relevant for the aquaculture industry; a variety of alternative oils, with different levels of LA content, can be used in feed formulation, but the possible resultant impact on *in vivo* n-3 LC-PUFA biosynthesis (and thus the final n-3 LC-PUFA content of cultured products) has not yet been established.

## Materials and Methods

### Ethics Statement

All animals and procedures used in this experimentation were approved by the Deakin University Animal Welfare Committee (A99-2010). All possible steps towards minimizing animal suffering were taken.

### Animals, Experimental Design and Sampling

Rainbow trout (*Oncorhynchus mykiss*) sourced from the Department of Primary Industries, Snob’s Creek hatchery (Victoria, Australia), were transported to Deakin University's Aquaculture Research Facility at the Warrnambool campus. Prior to the commencement of the experiment, fish were acclimatised to the experimental conditions for 2 weeks and maintained on a commercial trout diet (Skretting, Cambridge, Tasmania, Australia) ration at 2% of their body weight daily. The experiment was conducted in a closed loop, thermostatically and photoperiod-controlled recirculating aquaculture system (RAS). The system consisted of 18 (1000 L) rearing tanks (only 6 were used in the present study), with biological and physical filtration (drum filter with 60-µm screen; Hydrotech,Vellinge, Sweden), in-line oxygen enrichment and ultra violet (UV) disinfection. The system was maintained on a 12∶12 hour light:dark cycle. Temperature (15.0±1°C) and dissolved oxygen (9.1±.3 mg/L) were maintained at optimal conditions. The levels of metabolic waste, total ammonium and nitrite, were monitored bi-weekly using Aquamerck test kits (Merck, Darmstadt, Germany), and they remained below 0.50 and 0.05 mg L^−1^, respectively, for the duration of the trial.

Two iso-proteic and iso-lipidic experimental diets were formulated to contain 200 mg/g of lipid and 400 mg/g of protein, varying only in lipid source (**Table 1**). Three lipid sources (linseed oil, traditional sunflower oil and high oleic sunflower oil) were used and blended to obtain two experimental diets characterised by an abundant ALA content and identical overall fatty acid composition, with the exception of LA and oleic acid (OA, 18∶1n-9). The two experimental diets were named high linoleic acid diet (HLA) and low linoleic acid diet (LLA), and the only expected difference in their fatty acid composition was the relative content of these two fatty acids. The experimental diets were manufactured and stored as previously described [33].

**Table 1:** Formulation and proximate composition of the experimental diets.

	Experimental Diets^1^	
	LLA	HLA
***Diet formulation (g/kg)***		
**Basal Diet** 2	842.8	842.8
**Linseed oil**	78.6	78.6
**High Oleic acid Sunflower oil**	78.6	–
**Traditional Sunflower oil**	–	78.6
***Proximate composition (mg/g)***		
**Moisture**	61.6	56.0
**Protein**	407.2	414.7
**Lipid**	192.5	194.9
**Ash**	73.6	73.6
**NFE** 3	265.0	260.8
**Energy (MJ/Kg)** 4	21.8	22.0

1 Experimental diets abbreviations: LLA Low Linoleic Acid diet; HLA – High Linoleic Acid diet.

2 Basal diet composition (g/kg): fish meal 129.4; poultry by-product meal 318.5; soybean protein concentrate 124.4; blood meal 24.9; pre-gelatinised starch 209.0; mineral and vitamin mix 8; amino acid mix (methionine, lysine, aspartic acid and glutamic acid) 23.6; Celite® 5.

3 Nitrogen free extracts calculated by difference.

4 Calculated on the basis of 23.6, 39.5 and 17.2 kJ/g of protein, fat and carbohydrate, respectively.

Following an acclimation period, an initial sample of 10 fish (initial sample) were euthanized in excess anaesthetic (AQUI-S, 0.5 ml L^−1^) and stored at −20°C until subsequent analysis. 120 fish (mean weight 13.5±0.1 g) were randomly distributed into 6 tanks (20 fish per tank). Each of the tanks was then randomly assigned one of the two dietary treatments in triplicate (3 tanks per treatment; *n* = 3, *N* = 6). Fish were fed with one of the two experimental diets twice daily to apparent satiation at 0900 and 1600 h for an 88 day grow-out period. Feed consumption and mortalities were recorded throughout the duration of the trial. Faeces were collected from day 63 to 88, for subsequent digestibility estimation. At the completion of the grow-out phase, all fish were anesthetized and weighed, and 33 fish from each treatment (11 fish per tank) were randomly selected, euthanized in excess anaesthetic and stored at −20°C until subsequent analysis. The 11 sampled fish from each replicate (tank) were randomly split into 3 groups: the first group (4 fish) was used for biometry; the second group (4 fish) was used for chemical analysis of the whole body; and the third group (3 fish) was used for chemical analysis of fillet and liver. From this last group, an aliquot of ∼150 mg of liver tissue was taken immediately and snap frozen by immersion in liquid nitrogen for subsequent RNA isolation.

### Chemical Analyses, Nutrient Digestibility and Growth Performances

The chemical composition of the experimental diets, faeces and fish samples was determined via proximate composition analysis according to standard methods, as previously described [34]. Briefly, moisture was determined by drying samples in an oven at 80°C to a constant weight. Ash was determined by incinerating samples in a muffle furnace (Wit, C & L Tetlow, Australia) at 550°C for 18 h. Protein (Kjeldahl nitrogen: N×6.25) content was determined using an automated Kjeltech 2300 (Foss Tecator, Geneva, Switzerland). Lipid was determined using a cold extraction technique of Folch, *et al*., [35] as modified by Ways and Hanahan [36], with the only modification that dichloromethane was used to replace chloroform for safety reasons. Following lipid extraction, fatty acids were esterified into methyl esters using an acid-catalysed methylation method and then analysed by gas chromatography using an Agilent Technologies GC 7890A (Agilent Technologies, Santa Clara, California, USA) and published methods [37].

Faeces were collected for the evaluation of digestibility as previously described [34], and digestibility was determined by assessing acid insoluble ash (AIA), as specifically adapted to rainbow trout and previously described [38].

Standard formulae were used to assess growth, feed utilisation and other relevant parameters; these included initial and final average weight, total feed consumption, specific growth rate (SGR), food conversion ratio (FCR), dress-out percentage (DP%), hepatosomatic index (HSI%) and condition factor (K).

### Fatty Acid Metabolism, RNA Isolation and Gene Expression

The estimation of the apparent *in vivo* fatty acid metabolism was computed using the whole-body fatty acid balance method, as initially proposed and described by Turchini et al., [39] with further development [18], [40].

Total RNA was extracted from approximately 10 mg of liver tissues using TRI-reagent (Sigma) followed by phase separation with chloroform then precipitation with isopropanol. The RNA concentration and purity was determined using a Nanodrop 1000 spectrophotometer (Thermo Scientific). One µg of RNA was treated with DNAse 1 (Invitrogen) prior to reverse transcription using Superscript III Reverse Transcriptase (Invitrogen), according to manufacturer’s protocol. The synthesized first-strand cDNA (40 µL) was diluted to 80 µL using nuclease-free water and stored at −20°C. The expression of fatty acid Δ-6 desaturase and fatty acid elongase (Elovl5) mRNA was determined by semi-quantitative real-time PCR (qPCR). qPCR was performed in duplicate using a Rotor Gene RG 3000 (Corbett Research) in a 25 µL reaction containing 2 µL of diluted cDNA, the primer pair (100 nM of forward and reverse) and 12.5 µL of SYBR® Premix Ex Taq™ (Takara Bio) and a two-step reaction profile (94°C for 5 seconds, 60°C for 20 seconds). A melting curve analysis was performed at the end of 40 cycles to determine the specificity of the reaction. The mRNA expression was normalized by the ratio of the threshold cycle (Ct) value to the concentration of the single stranded cDNA and is given by 2-ΔΔCt. The concentration of single-stranded cDNA was quantified against oligonucleotide standards in an assay using a Quant-iT OliGreen ssDNA reagent and kit (Invitrogen). Specific primer pairs were designed for rainbow trout based on the gene sequences available in GenBank (http://www.ncbi.nlm.nih.gov): Δ-6 desaturase (accession no. NM001124287) forward; 5′-ACCTAGTGGCTCCTCTGGTC-3′, reverse; 5′-CAGATCCCCTGACTTCTTCA-3′) and elongase 5 (accession no. AY605100) forward; 5′- TCAACATCTGGTGGTTCGTCAT-3′, reverse; 5′- TGTTCAGGGAGGCACCAAAG-3′) using Primer Express (ver. 3, Applied Biosystems). Amplification of the correct cDNA was confirmed by sequencing.

### Statistical Analysis

All data were reported as mean ± standard error (*N* = 3, *N* = 6). After confirmation of normality and homogeneity of variance, data was subjected to an independent *T*-test. Significance was accepted at *P*<0.05, and *P* values were reported as * *P*<0.05, ** *P*<0.01 and *** *P*<0.001. All statistical analyses were performed using SPSS v17.0 (SPSS Inc., Chicago, IL, USA).

## Results

During the 88 day feeding trial, no mortality was recorded. Fish grew up to over 15-fold their initial body weight, and no statistically significant differences were recorded for any of the growth and feed utilisation parameters, biometrical data or nutrient digestibility (**Table 2**).

**Table 2:** Growth and feed utilization parameters, biometrical data and apparent digestibility coefficients of rainbow trout fed the experimental diet for 88 days.

	Experimental treatments^1^		
	LLA	HLA	*P-value*
*Growth and feed utilization*			
Initial weight (g)	13.4±0.8	13.6±0.3	*ns*
Final weight (g)	206.7±6.5	193.9±10.3	*ns*
Total feed consumption(g/fish)	226.3±9.0	228.4±10.3	*ns*
SGR^2^	3.1±0.0	3.0±0.1	*ns*
FCR^3^	1.1±0.0	1.2±0.1	*ns*
*Biometrical parameters*			
DP%^4^	82.86±0.07	83.01±1.11	*ns*
HSI%^5^	1.81±0.06	1.77±0.02	*ns*
K^6^	1.68±0.04	1.66±0.01	*ns*
*Nutrients digestibility (%)*			
ADC^7^ (Dry matter)	80.5±0.7	82.7±1.3	*ns*
ADC (Lipid)	94.8±0.3	94.9±0.4	*ns*
ADC (Protein)	88.3±0.2	90.0±0.8	*ns*
*Fatty acid digestibility (%)*			
ADC (18∶1n-9)	96.34±0.42	95.99±0.25	*ns*
ADC (18∶2n-6)	97.46±0.21	97.50±0.15	*ns*
ADC (18∶3n-3)	98.21±0.18	98.03±0.14	*ns*

Data are expressed as mean ± s.e.m., *n*  = 3; *N*  = 6. P-value: *ns* = not significant;

* , ** and *** indicate P<0.05, <0.01 and <0.001, respectively.

1 See table 1 for experimental diets abbreviation.

2 SGR = Specific Growth Rate.

3 FCR = Food conversion ratio.

4 DP% =  dress-out percentage.

5 HSI% = hepatosomatic index.

6 Fulton's condition factor.

7 ADC = Apparent Digestibility Coefficients.

The fatty acid composition of the two experimental diets and of the trout fillets at the start and end of the feeding trial is presented in **Table 3**. The two diets had an almost identical overall fatty acid composition, with the only difference being relative to their respective content of 18∶1n-9 and 18∶2n-6; the LLA diet contained 466.3 and 105.1 (mg/g lipid) and the HLA diet contained 255.8 and 307.6 (mg/g lipid) of the two fatty acids, respectively. At the end of the feeding trial, the trout fillet showed statistically significant differences for the content of MUFA and n-6 PUFA (particularly 18∶1n-9, 18∶1n-7, 18∶2n-6, 18∶3n-6, 20∶1n-9, 20∶2n-6, 20∶3n-6, 20∶4n-6, 22∶1n-9 and 22∶2n-6), but there were no differences found for any of the n-3 PUFA.

**Table 3:** The fatty acid composition (mg/g lipid) of experimental diet, initial fish fillets and rainbow trout fillet fed the experimental diets for 88 days.

	Experimental diets^1^			Experimental treatments^1^		
	LLA	HLA	*Initial*	LLA	HLA	*P-value*
14∶0	4.9	4.8	20.0	7.3±0.2	6.9±0.3	
14∶1n-5	0.5	0.4	–	–	–	
15∶0	0.7	0.6	2.3	0.4±0.2	0.1±0.1	
16∶0	95.2	104.3	171.8	114.1±1.6	114.5±1.0	
16∶1n-7	16.9	16.4	35.1	27.2±1.2	22.2±2.1	
18∶0	42.0	43.7	48.6	38.7±0.4	40.6±0.8	
18∶1n-9	466.3	255.8	173.7	387.6±5.2	225.5±3.7	***
18∶1n-7	10.1	10.5	23.3	12.8±0.2	11.6±0.3	*
18∶2n-6	105.1	307.6	56.5	70.9±1.2	196.1±3.9	***
18∶3n-6	0.4	0.2	1.1	2.0±0.1	4.3±0.4	**
18∶3n-3	196.1	198.0	10.4	96.8±2.0	95.7±2.8	
18∶4n-3	0.9	0.7	4.8	12.0±0.1	11.0±0.7	
20∶0	2.1	2.0	1.0	1.5±0.0	1.6±0.1	
20∶1n-11	0.7	0.7	–	0.0±0.0	0.0±0.0	
20∶1n-9	3.4	2.6	7.9	10.5±0.2	7.2±0.1	***
20∶2n-6	0.4	0.4	3.3	3.4±0.1	9.3±0.3	***
20∶3n-6	–	–	3.1	3.6±0.1	8.3±0.6	**
20∶4n-6	1.8	1.8	11.5	3.7±0.1	6.1±0.2	***
20∶3n-3	–	–	–	4.4±0.1	4.4±0.2	
20∶4n-3	0.6	0.5	9.2	6.1±0.3	5.8±0.6	
20∶5n-3	7.0	6.2	57.9	9.7±0.6	8.7±0.1	
22∶0	2.1	2.0	1.0	1.5±0.0	1.5±0.1	
22∶1n-11	–	–	2.5	0.4±0.2	0.2±0.2	
22∶1n-9	0.7	0.8	1.0	1.4±0.0	0.8±0.1	**
22∶2n-6	–	–	1.0	0.5±0.2	1.4±0.1	*
22∶4n-6	0.4	0.5	4.1	0.8±0.2	1.3±0.1	
22∶3n-3	0.8	0.6	2.4	0.8±0.3	2.0±0.7	
22∶5n-3	1.5	1.5	22.1	4.0±0.1	3.7±0.0	
22∶6n-3	7.2	6.9	197.3	37.8±1.6	41.4±2.4	
Total Fatty acids^2^	967.4	969.2	872.8	859.6±11.9	832.3±5.8	
SFA	146.9	157.4	244.6	163.5±2.2	165.4±1.3	
MUFA	498.5	287.2	243.5	439.8±6.4	267.5±6.3	***
PUFA	322.1	524.6	384.7	256.3±4.7	399.5±10.5	***
n-6 PUFA	108.1	310.2	80.7	84.7±1.5	226.8±4.6	***
n-6 LC-PUFA	2.6	2.6	23.0	11.8±0.7	26.4±1.0	***
n-3 PUFA	214.0	214.4	304.1	171.6±3.1	172.7±6.0	
n-3 LC-PUFA	17.1	15.7	288.9	62.8±2.1	66.0±3.7	

Data are expressed as mean ± s.e.m., *n*  = 3; *N*  = 6. P-value: *ns* = not significant; *, ** and *** indicate P<0.05, <0.01 and <0.001, respectively (diets and initial fish not included in the statistical test).

1 See table 1 for experimental diets and treatments abbreviation.

2 Fatty acid class abbreviations: SFA: saturated fatty acids; MUFA: monounsaturated fatty acids; PUFA: polyunsaturated fatty acids; LC-PUFA, long chain polyunsaturated fatty acids (<20 carbon atoms); n-6: omega-6 fatty acids; n-3: omega-3 fatty acids.

**Figure 1:**
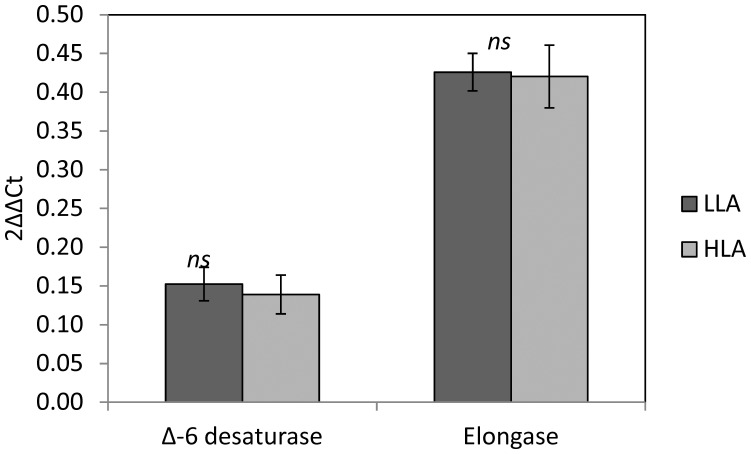
Differential gene expression of fatty acid Δ-6 desaturase and elongase in liver of rainbow trout fed the two different experimental diets. Data presented as mean ± s.e.m. (*n* = 3; *N* = 6). No statistically significant differences were observed.

In trout liver, the transcription rates of fatty acid Δ-6 desaturase and fatty acid elongase were not affected by the dietary treatment (**Figure 1**). The overall apparent *in vivo* fatty acid enzymatic activities are reported in **Table 4**, with significant differences found for elongation, desaturation (Δ-6 and Δ-5) and β-oxidation of n-6 PUFA, and elongation and Δ-9 desaturation of SFA and MUFA. No significant differences were recorded for any enzymatic activities on n-3 PUFA or for β-oxidation of SFA and MUFA.

**Table 4:** The apparent *in vivo* enzymatic activities (nmol/g/day), including *de novo* production, β-oxidation, desaturation and elongation of total fatty acids grouped per main classes, in rainbow trout fillet fed the experimental diets for 88 days and deduced by the whole body fatty acid balance method.

	Experimental diets^1^		*P-Value*
	LLA	HLA	
***SFA and MUFA*** 2			
*De novo* production	520±78	149±135	*ns*
Total elongation	666±76	202±142	*
Total β-oxidation	1,161±199	1,213±514	*ns*
Total Δ-9 desaturation	269±17	93±60	*
***n-6 PUFA***			
Total elongation	52±14	134±11	*
Total β-oxidation	513±21	2,101±345	*
Total Δ-6 desaturation	54±15	175±11	**
Total Δ-5 desaturation	5±5	41±5	**
***n-3 PUFA***			
Total elongation	904±32	775±69	*ns*
Total β-oxidation	1,047±80	1,440±247	*ns*
Total Δ-6 desaturation	718±22	668±60	*ns*
Total Δ-5 desaturation	256±10	220±30	*ns*

Data are expressed as mean ± s.e.m., *n*  = 3; *N*  = 6. P-value: *ns* = not significant;

* , ** and *** indicate P<0.05, <0.01 and <0.001, respectively.

1 See table 1 for experimental diets and treatments abbreviation.

2 See table 3 for fatty acid classes abbreviations. ^2^Fatty acid classes abbreviations: SFA: saturated fatty acids; MUFA: monounsaturated fatty acids; n-6 PUFA: omega-6 polyunsaturated fatty acids; n-3 PUFA: omega-3 polyunsaturated fatty acids.

**Figure 2:**
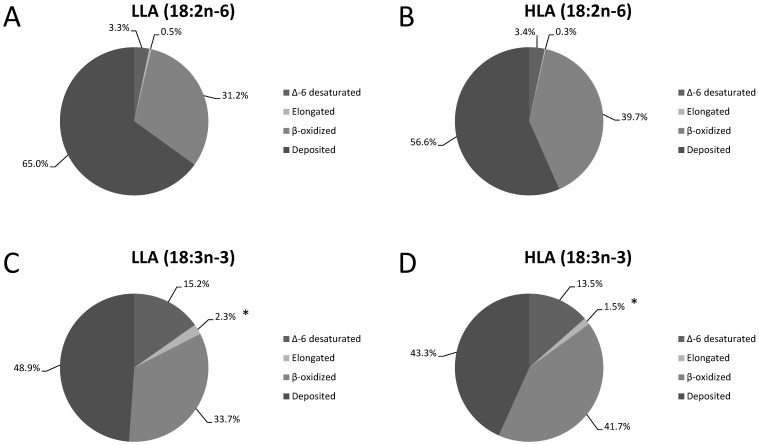
The apparent *in vivo* fate of 18∶2n-6 or 18∶3n-3 towards direct elongation to 20∶2n-6 or 20∶3n-3, Δ-6 desaturation to 18∶3n-6 or 18∶4n-3, β-oxidation or deposition as is, expressed as percentage of dietary net intake, in rainbow trout fed the two experimental diets (LLA and HLA) and deduced by the whole-body fatty acid balance method. (**A**) *in vivo* fate of 18∶2n-6 in trout fed LLA; (**B**) *in vivo* fate of 18∶2n-6 in trout fed HLA; (**C**) *in vivo* fate of 18∶3n-3 in trout fed LLA; (**D**) *in vivo* fate of 18∶3n-3 in trout fed HLA. In each graph, data represent mean values (*n*  = 3; *N*  = 6), and the only statistically significant difference recorded was for the percentage of 18∶3n-3 directly elongated to 18∶4n-3 in trout fed LLA or HLA (*P<0.05).

The metabolic fate of 18∶2n-6 or 18∶3n-3 towards direct elongation, Δ-6 desaturation, β-oxidation or direct deposition (expressed as percentage of dietary net intake) in trout fed the two experimental diets is reported in **Figure 2**. For both treatments, the majority of both dietary 18∶2n-6 and 18∶3n-3 was directly deposited (from 43.3 to 65%), and a relatively large amount was β-oxidised (from 31.2 to 41.7%). Only about 3.3% of 18∶2n-6 was Δ-6 desaturated, whilst up to 15% of dietary 18∶3n-3 was Δ-6 desaturated. The only statistically significant difference recorded was for the direct elongation of 18∶3n-3 towards the production of 20∶3n-3, which was significantly higher in LLA compared to HLA.

**Figure 3:**
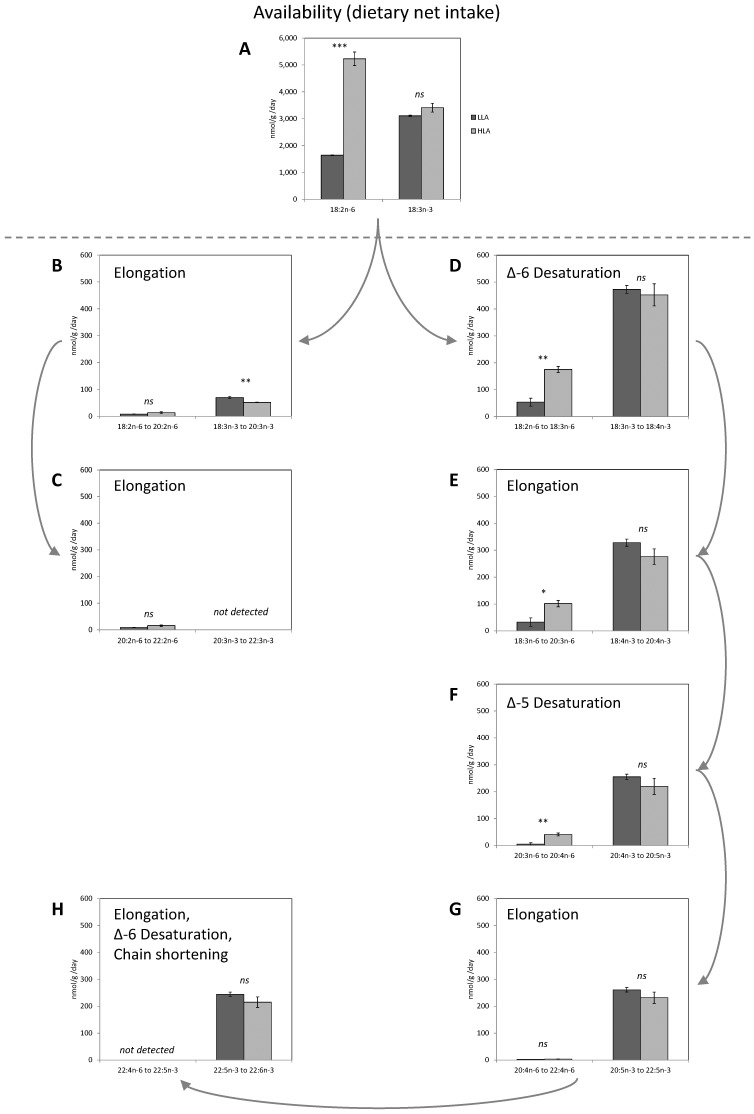
The apparent *in vivo* 18∶2n-6 and 18∶3n-3 bioconversion activity (nmol/g/day) in rainbow trout fed the two experimental diets (LLA and HLA) and deduced by the whole-body fatty acid balance method. Graphs are reported following the PUFA bioconversion pathway, from (**A**) substrate availability (dietary net intake); towards i) the dead end pathway (on the left): (**B**) elongation of 18∶2n-6 and 18∶3n-3; (**C**) elongation of 20∶2n-6 and 20∶3n-3; and ii) the LC-PUFA biosynthetic pathway(on the right): (**D**) Δ-6 desaturation of 18∶2n-6 and 18∶3n-3; (**E**) Elongation of 18∶3n-6 and 18∶4n-3; (**F**) Δ-5 desaturation of 20∶3n-6 and 20∶4n-3; (**G**) Elongation of 20∶4n-6 and 20∶5n-3; (**G**) Elongation, Δ-6 desaturation and chain shortening of 22∶4n-6 and 22∶5n-3. In each graph, bars represent mean ± s.e.m., *n*  = 3; *N*  = 6. P-value: *ns* = not significant; *, ** and *** indicate P<0.05, <0.01 and <0.001, respectively.

In rainbow trout fed the two experimental diets (LLA and HLA), the apparent *in vivo* bioconversion activities (nmol/g/day) for all the individual steps along the fatty acid bioconversion pathway, and deduced by the whole-body fatty acid balance method, are reported in **Figure 3**. With regards to the availability of the two initial fatty acid substrates (dietary net intake of 18∶2n-6 and 18∶3n-3), a large and statistically significant difference was recorded for 18∶2n-6, whilst no difference was recorded for 18∶3n-3 (**Figure 3A**). A statistically significant higher elongation of 18∶3n-3 toward the production of 20∶3n-3 was recorded in LLA fed fish, compared to HLA fed fish (**Figure 3B**), whilst no statistically significant differences were recorded for any bioconversion step of 18∶3n-3 towards the final production of 22∶6n-3 (**Figures 3D–H**). On the other hand, for the n-6 PUFA series, statistically significant differences were recorded for the Δ-6 desaturation of 18∶2n-6 towards the production of 18∶3n-6 (**Figure 3D**), the elongation of 18∶3n-6 towards the production of 20∶3n-6 (**Figure 3E**) and the Δ-5 desaturation of 20∶3n-6 towards the production of 20∶4n-6 (**Figure 3D**).

## Discussion

The two experimental diets were formulated to provide optimal nutrition for trout, differing only in their fatty acid composition. In the present trial, we have shown that fish fed either experimental diets showed optimal growth performances, and no effects of the diet were observed on growth performances, feed utilisation parameters, biometric data and nutrient digestibility, which is largely consistent with several previous studies [3].

As is well-documented, the fatty acid composition of fish tissues is reflective of that present in their diet [1]–[3], [7]. In this study, it was shown that the fillet of fish fed LLA (containing higher amounts of 18∶1n-9) recorded a higher level of 18∶1n-9 in the fillet, and also other fatty acids directly derived from the *in vivo* bioconversion of this fatty acid, such as 20∶1n-9 and 22∶1n-9. In addition, the fillet of fish fed HLA (containing higher amounts of 18∶2n-6) recorded a higher level of 18∶2n-6, and also other fatty acids directly derived from the *in vivo* bioconversion of this fatty acid, such as 18∶3n-6, 20∶2n-6, 20∶3n-6 and 20∶4n-6. These observations clearly suggest that the dietary fatty acid composition is critical in determining the fatty acid composition of fish tissues, and that *in vivo* fatty acid metabolism can have a measurable effect.

The specific objective of this study was to assess the possible existence of substrate competition for the Δ-6 desaturase enzyme. Previously, it has been documented that the transcription rates of genes encoding the enzymes involved in the LC-PUFA biosynthetic pathway can be affected by the presence or absence of dietary LC-PUFA [11], [28], [30]. However, in the present study, both experimental diets contained identical concentrations of LC-PUFA, and thus, similar genes transcription rates should have been expected. Accordingly, there were no differences in the hepatic fatty acid Δ-6 desaturase and fatty acid elongase mRNA transcription rates between the two dietary treatments. This is an important observation, as any possible difference in the apparent *in vivo* enzymatic activity cannot be attributed to different gene transcription rates.

In the present study, we have recorded significant effects of the dietary treatment on the apparent *in vivo* bioconversion of MUFA and n-6 PUFA, and this is consistent with a previous observation that these metabolic pathways are substrate limited in teleosts [31]. Thus, fish fed the LLA diet (receiving more dietary 18∶1n-9) recorded an increased bioconversion of this fatty acid, whilst fish fed the HLA diet (richer in 18∶2n-6) recorded increased activities in the various bioconversion steps of n-6 PUFA.

The most important finding of the present study was, that independently from the availability of 18∶2n-6, the bioconversion of 18∶3n-3 towards longer chain and more unsaturated fatty acid was almost completely unaffected. The only observed significant difference was for the amount of 18∶3n-3 directly elongated towards 20∶3n-3 (commonly referred to as “the dead end pathway”), which was higher in fish fed the LLA. This clearly supports the existence of a direct substrate competition between 18∶2n-6 and 18∶3n-3 for accessing the fatty acid elongase. On the other hand, no differences were observed in the Δ-6 desaturation of 18∶3n-3, and also in any of the subsequent bioconversion steps towards the final production of 22∶6n-3.

As mentioned earlier, the paradigm that the two substrates (18∶2n-6 and 18∶3n-3) are in direct competition for accessing Δ-6 desaturase, the first of the bioconverting enzymes for LC-PUFA biosynthesis, has been well-documented and demonstrated for mammals [24]–[27]; consequently, it has been accepted by the scientific community. However, it has been previously suggested that the fatty acid Δ-6 desaturase may behave quite differently in teleosts [28]. The results of our study strongly suggest that in rainbow trout there is no apparent competition between the two substrates (18∶2n-6 and 18∶3n-3) for accessing Δ-6 desaturase. Thus, a specific paradigm shift should be considered from ALA and LA being in competition for accessing Δ-6 desaturase enzyme, to ALA and LA being Δ-6 desaturated independently from the presence and abundance of each other.

Δ-6 desaturase is known to be a single enzyme required twice within the LC-PUFA biosynthetic pathway, and acting on both n-6 and n-3 PUFA [10]–[13], [28]. Accordingly, this metabolic pathway has also been well-established for rainbow trout [41], [42]. Nevertheless, the findings of our study have shown that substrate competition is not a limiting factor for the activity of this enzyme, and are in agreement with our previous observation that the provision of increased dietary 18∶4n-3 and 18∶3n-6 did not permit for increased activity on the 24∶5n-3 substrate [32]. Therefore, as reported in this study, “paradoxically, it appears that the activity of the same enzyme on the different substrates is an independent process”.

Admittedly, even if no statistically significant differences were recorded, when compared to fish fed LLA, fish fed the HLA diet received *a* numerically higher amount of dietary 18∶3n-3, and recorded a numerically lower mean apparent *in vivo* bioconversion activity on this fatty acid. This may suggest the existence of a minor substrate competition, but this can only be considered as being trivial as the actual *in vivo* n-3 LC-PUFA biosynthesis was statistically unaffected by the amount of 18∶2n-6 provided in the diet. This trend was further observed in all 18∶3n-3 derivatives along the bioconversion pathway. Notably, the difference in percentage of substrate desaturated at the final Δ-6 desaturation step to 22∶6n-3 did not increase as would have been expected under the competing substrates paradigm. This lack of reduction in production of 22∶6n-3 as a result of increased LA further strengthens the argument of no evident substrate competition between n-3 and n-6 for access to Δ-6 desaturase in rainbow trout.

### Conclusions

In the present study, we have shown that in rainbow trout there is no evidence of substrate competition for accessing the fatty acid Δ-6 desaturase. This finding is envisaged to be useful towards increasing our understanding of fatty acid metabolism in teleosts, which seems to be considerably different from that of mammals. From a more practical point of view, this finding can also be considered as being applicable in the aquaculture industry, since when formulating aquafeed towards maximising the potential *in vivo* n-3 LC-PUFA biosynthesis in cultured fish, priority should be given to using alternative oils rich in 18∶3n-3 irrespective of any concern as to their 18∶2n-6 content.
